# Hybrid Geopolymers from Fly Ash and Polysiloxanes

**DOI:** 10.3390/molecules24193510

**Published:** 2019-09-27

**Authors:** Giuseppina Roviello, Laura Ricciotti, Antonio Jacopo Molino, Costantino Menna, Claudio Ferone, Raffaele Cioffi, Oreste Tarallo

**Affiliations:** 1 Dipartimento di Ingegneria, Università di Napoli ‘Parthenope’, Centro Direzionale, Isola C4, 80143 Napoli, Italy; giuseppina.roviello@uniparthenope.it (G.R.); antoniojacopo.molino@uniparthenope.it (A.J.M.); claudio.ferone@uniparthenope.it (C.F.); raffaele.cioffi@uniparthenope.it (R.C.); 2 INSTM Research Group Napoli Parthenope, National Consortium for Science and Technology of Materials, Via G. Giusti, 9 50121 Firenze, Italy; 3 Dipartimento di Strutture per l’Ingegneria e l’Architettura, Università degli Studi di Napoli “Federico II”, 80125 Napoli, Italy; costantino.menna@unina.it; 4 Dipartimento di Scienze Chimiche, Università degli Studi di Napoli “Federico II”, Complesso Universitario di Monte S. Angelo, via Cintia, 80126 Napoli, Italy

**Keywords:** hybrid geopolymer, geopolymer, fly ash, polysiloxane, microtomography, morphology, mechanical properties

## Abstract

The preparation and characterization of innovative organic-inorganic hybrid geopolymers, obtained by valorizing coal fly ash generated from thermoelectric power plants, is reported for the first time. These hybrid materials are prepared by simultaneously reacting fly ash and dimethylsiloxane oligomers at 25 °C in a strongly alkaline environment. Despite their lower density, the obtained materials are characterized by highly improved mechanical properties when compared to the unmodified geopolymer obtained without the use of polysiloxanes, hence confirming the effectiveness of the applied synthetic strategy which specifically aims at obtaining hybrid materials with better mechanical properties in respect to conventional ones. This study is an example of the production of new materials by reusing and valorizing waste raw resources and by-products, thus representing a possible contribution towards the circular economy.

## 1. Introduction

The great interest in hybrid materials is due to their key role in the development of advanced functional materials capable of providing peculiar chemical and physical properties. In fact, the synergistic interactions between organic and inorganic phases result in new materials with special properties that do not simply arise from the sum of the contributions of both initial constituents [1,2,3]. Indeed, most of these systems typically find application in fields such as energy, health, environment, nanotechnology, packaging, and building.

Organic-inorganic hybrid materials can be synthesized following different strategies [4]: (i) The insertion of organic compounds in a previously formed inorganic host (e.g., intercalation in porous or lamellar hosts such as clays and layered silicates); (ii) the in situ generation or nanometric dispersion of inorganic compounds in a polymeric network; (iii) the reaction of inorganic compounds around the organic phase as the host; (iv) the reaction of organic compounds around inorganic core; and (v) the simultaneous reactions of polymerization and/or the co-reticulation of both organic and inorganic networks [4].

Among the different types of organic-inorganic hybrid materials, a particularly interesting area, even if not sufficiently explored, is that geopolymer-based ones [5].

Geopolymers are amorphous materials that can be obtained from the alkaline activation of an aluminosilicate source (e.g., metakaolin) and an alkali–silicate solution. Geopolymers are characterized by interesting mechanical properties such as low shrinkage, thermal stability, freeze-thaw resistance, chemical and fire resistance, long term durability, and recyclability. Thanks to these features, the application of geopolymers in fields related to refractory materials, binders for hazardous or radioactive wastes encapsulation, high-tech ceramics, or adhesives have been proposed [6]. Geopolymers could be also considered promising materials to substitute traditional cement-based binders like ordinary Portland cement (OPC) in different applications such as fireproof barriers, materials for high temperatures, tooling, and moldings [7]. Moreover, with respect to OPC, geopolymers could significantly reduce CO_2_ emissions by replacing their starting raw materials with industrial or agricultural waste which presents a high concentration of aluminosilicates such as fly or bottom ash, rice husk or red mud [8].

However, one important requirement that geopolymer-based materials need to meet for structural applications is the reduction of their brittleness, thus avoiding damage or catastrophic failure during service conditions.

In order to find a possible solution to this problem, in the last few years, we have succeeded in the obtainment of novel geopolymer-based composites and hybrid materials [9,10,11,12,13,14,15,16,17,18,19,20,21,22]. In particular, we proposed an innovative synthetic route for the obtainment of new geopolymer-based organic-inorganic materials by means of the simultaneous reticulation of an organic phase (e.g., epoxy resin precursors or siloxane oligomers) and an inorganic one (e.g., metakaolin) [11,16]. Within this framework, the most promising results were obtained by reacting metakaolin and an alkali–silicate solution with mixtures of dialkylsiloxane oligomers [16]. In this last case, the co-reticulation reaction allowed for the obtainment of a single-phase amorphous hybrid material made of an interpenetrated network of Si–O–Si units deriving both from the inorganic source and the siloxane oligomers. In this network, organic and inorganic moieties are linked by chemical bonds, giving rise to a class II hybrid material [23]. This achievement was possible thanks to the chemical similarity of the siloxane units with the geopolymer structure and thanks to their capability to polymerize in an alkaline environment, i.e., under the same conditions promoting the geopolymerization reaction. In such a way, during the simultaneous polycondensation reaction, the formation of oxygen bridges between silicon atoms deriving from the aluminosilicate source and those deriving from polysiloxane was allowed. It is worth pointing out that these new hybrid materials turned out to have significantly enhanced compressive strength and stiffness compared to mechanical properties of unmodified (e.g., without the organic component) geopolymers or to those of organic-inorganic composites with similar chemical compositions [16].

Such hybrid materials become even more interesting if prepared by using, as a starting raw material, industrial or agricultural wastes (such as fly ash, slag blast furnace or rice husk ash). In this way, as already reported in the literature for conventional geopolymers, [24,25], the critical drawbacks associated with raw material supply for mass production could be overcome. In particular, fly ash (FA) is a fine powder by-product generated in very large amounts in coal-fired power stations. FA is typically made up of small glass spheres containing amorphous silico-aluminous phase that can be successfully used in the production of value-added materials, such as geopolymers, with the aim of recycling waste and, at the same time, reducing CO_2_ emissions [24,25]. However, FA-based geopolymers also typically exhibit poor mechanical properties. 

In this paper, aiming to improve the mechanical and physical properties of FA-based geopolymers, we investigated the possibility to obtain fly ash-based hybrid geopolymers by applying the synthetic approach described in [16], that is the simultaneous chemical reaction among the silico-aluminous phase present in fly ash and siloxane units present in polysiloxane oligomers. In this way, hybrid geopolymer-based materials with a lower cost, in terms of economic and environmental impacts with respect to those obtained from metakaolin, were obtained. The samples were characterized from a structural and morphological point of view. Mechanical properties were investigated as well.

## 2. Results and Discussion

### 2.1. Sample Preparation

**Figure 1:**
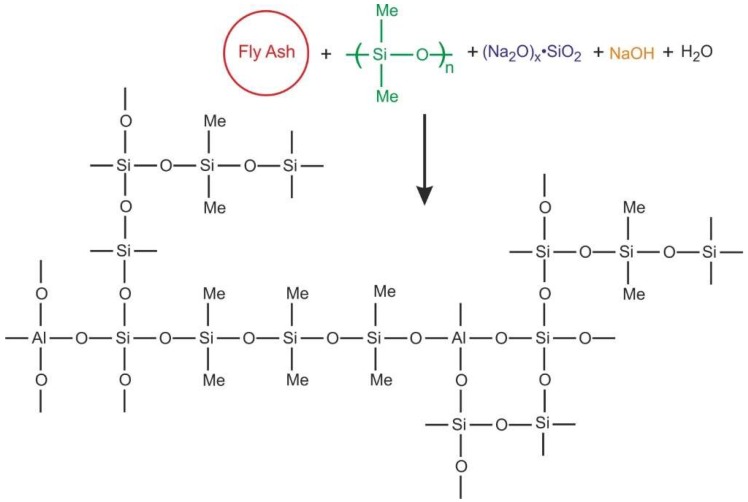
Schematic representation of the possible formation of bonds between dimethylpolysiloxane and aluminosilicate units during the polycondensation process that allows for the obtainment of a hybrid material according to the synthetic procedure described in [16]. The scheme only represents a possible situation that highlights the chemical bond formation between the organic and inorganic moieties in the final hybrid material since fly ash (FA)-based alkali-activated binders are often highly heterogeneous due to the variable chemical and physical nature of the particles present in the FA used as a precursor and the complex reactions taking place during alkali activation.

Hybrid FA-based polysiloxane-geopolymer samples (GSyl-FA) were prepared by applying a synthetic approach earlier developed by some of us [16], consisting of the concurrent reaction in mild conditions of a commercial oligomeric dimethylsiloxane mixture with FA dispersed in a silicate solution. In these conditions, both the polycondensation reaction of FA and the polymerization of siloxane units take place at the same time, and so, as already shown for the analogous metakaolin-based systems [16], the formation of chemical links between the reactive FA-based geopolymeric suspension and the dimethylsiloxane mixtures is likely to occur, thus forming a chemical crosslink between these two components (schematically represented in Figure 1). Additionally, in the present case, it is reasonable to suppose that class II hybrid materials can be obtained. It is worth pointing out that, very recently, our synthetic approach was successfully applied to obtain hybrid organic-inorganic geopolymeric cementitious materials utilizing fly ash and rice husk [26].

### 2.2. X-Ray Diffraction Characterization

**Figure 2:**
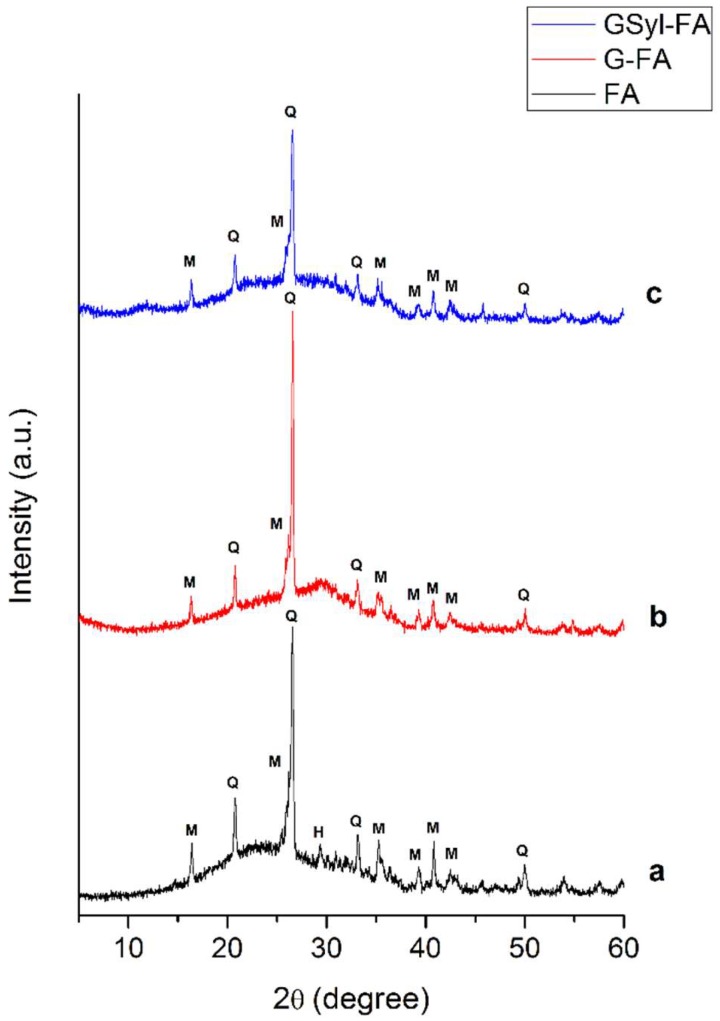
X-ray powder diffraction patterns of (**a**) fly ash (FA; black line); (**b**) the unmodified geopolymer (G-FA; red line) and (**c**) the hybrid fly ash-based polysiloxane-geopolymer (GSyl-FA; blue line). H = hematite; M = mullite; and Q = quartz.

Figure 2 shows the XRD patterns of the FA, the cured, unmodified FA-based geopolymer (G-FA) and the hybrid sample (GSyl-FA) containing 10 wt % of polysiloxane resin. The diffraction pattern of the fly ash (Figure 2a) was characterized by a wide and diffused hump in the interval range of 15–38 degrees of 2θ, with a maximum at 2θ ≈ 25. Minor crystalline phases (the degree of crystallinity was less than 20%) such as quartz (JCPDS 01-070-2517), mullite (JCPDS 01-076-2579) and hematite (JCPDS 00-013-0534) could be identified. This amorphous halo shifted towards slightly higher angular values (maximum at 2θ ≈ 25) in the G-FA sample, indicating the formation of an alkaline aluminosilicate hydrate gel (N–A–S–H) with a 3D amorphous structure [27]. The crystalline phases detected in the FA raw material, such as quartz and mullite, remained substantially unaltered according to the fact that in fly ash, only the amorphous aluminosilicate component was reactive for the geopolymerization reaction [28]. Finally, in regard to the hybrid specimen, the X-ray diffraction pattern was very similar to that of the FA-based geopolymer because the added amorphous organic phase did not significantly alter the diffraction pattern. 

### 2.3. Microstructural Analysis 

#### 2.3.1. Characterization of Fly Ash

**Figure 3:**
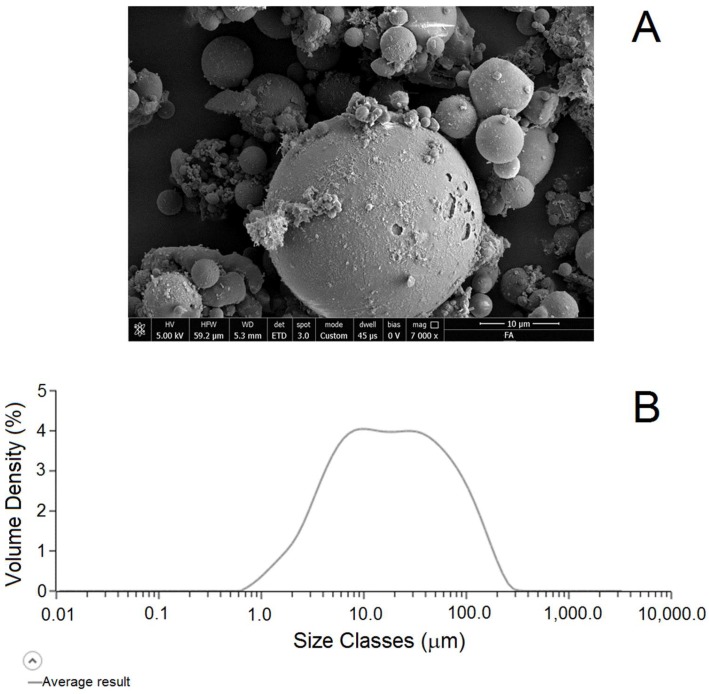
(**A**) Scanning electron microscope micrograph (magnification 7000×) and (**B**) particle size distribution (volume density vs. size) of the used fly ash.

Figure 3 shows a representative SEM micrograph (Figure 3A) and particle size distribution (Figure 3B) of the used fly ash. It is apparent that, in good agreement with what has already been reported in the literature, [29,30] fly ash consisted mostly of glassy cenospheres with a diameter varying in the range of 10–40 μm. In particular, the shape of the size distribution curve (Figure 3B) suggests a sort of bimodal distribution of the particle sizes, with a first maximum centered around 10 μm (that was likely to be the most common diameter of the fly ash particle, see Figure 3A) and a second maximum centered around 40 μm, probably due to particle aggregates. The D10 was 3.36 μm, the D50 was 17.7 μm, and the D90 was 92.6 μm.

#### 2.3.2. Microstructural Analysis of G-FA and GSyl-FA 

**Figure 4:**
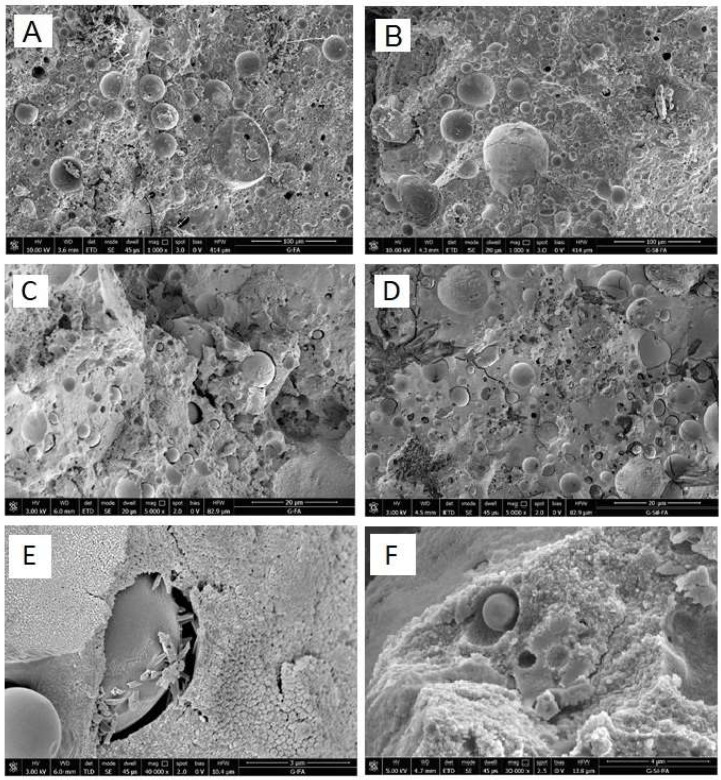
Scanning electron microscope (SEM) micrographs of G-FA (on the left, at different magnifications); of GSyl-FA (on the right, at different magnifications): (**A**,**B**) at 1000 magnifications; (**C**,**D**) at 5000 magnifications; (**E**,**F**) at 35,000 magnifications.

SEM micrographs of the freshly obtained fractured surfaces of the G-FA and GSyl-FA samples are reported in Figure 4. The morphologies of the G-FA and GSyl-FA samples appeared rather similar (Figure 4A–D). First, both examined specimens showed quite compact structures with only minor micro-cracks, likely due to the cutting of the specimens (Figure 4C,D). Moreover, both the G-FA and GSyl-FA samples showed a continuous structure in which it was possible to identify unreacted fly ash particles well-dispersed in the geopolymer matrix (Figure 4A,B). In addition, in the GSyl-FA sample, no phase segregated domains (that could be originated by the presence of the organic component) were detectable (Figure 4F). This fact suggests the formation of a strongly interpenetrated network between the inorganic and the polysiloxane component that could have been a consequence of the formation of chemical bonds between the aluminosilicate and siloxane components [16,26]. 

As a common feature of both samples, their morphology could be described as a three-dimensional network characterized by the presence of very small voids representing a diffuse porosity. This morphology could be attributed to the geopolymerization process. In fact, when the geopolymeric reactive suspension is obtained by mixing a sodium silicate solution and sodium hydroxide with fly ash as aluminosilicate source, the latter is hydrolyzed by the alkaline environment, leading to the formation of aluminate and silicate species [3]. Accordingly, silicate, aluminosilicate and aluminate products are formed, and a complex equilibrium between them is established [31]. The geopolymerization reaction proceeded in this strongly alkaline environment, leading to the formation of a gel due to the condensation of the oligomers into a large network. After gelation, a reorganization process of the system occurred, leading to a three-dimensional aluminosilicate network consisting of alternating Si and Al tetrahedrons with bridge oxygen atoms and alkali cations [3,32]. According to this geopolymerization mechanism, the observed porosity (Figure 4A,B) could be attributed to the removal of water molecules during the curing process of the geopolymer samples [33,34].

Moreover, SEM images at higher magnifications (Figure 4E,F) of the geopolymer matrixes of G-Fa and GSyl-FA clearly showed the presence of nodules with an average diameter of about 40–50 nm, analogous to those observed in geopolymer specimen obtained according to very similar experimental conditions from metakaolin as aluminum–silicate source [16].

Further data on the internal structure of the examined samples were obtained by means of micro-computed tomography. The knowledge of the fine microstructure allows for, in turn, the explanation of the macroscopic behavior of materials. In particular, microtomography allows for the highlighting of the presence and the distribution of cracks, inhomogeneous regions, or particles that make the material a non-continuum system, worsening its mechanical properties. Actually, FA-based alkali-activated binders are often highly heterogeneous due to the variable chemical and physical nature of the particles present in the fly ash used as a precursor, as well as the complex reactions taking place during alkali activation. Moreover, a deep analysis of the porosity is crucial for establishing its durability in order to explain the behavior of the material when it is subjected to the diffusion of aggressive substances, in particular acids, carbonates, or chlorides. 

**Figure 5:**
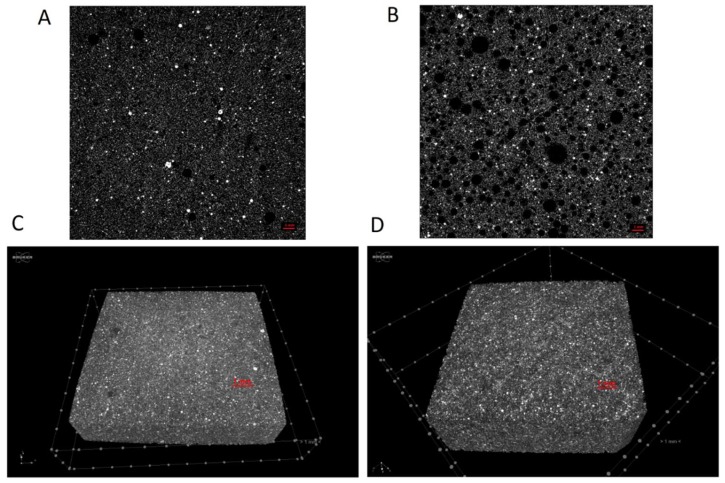
2D (**A**,**B**) and 3D (**C**,**D**) slice images obtained by X-ray microtomography of unmodified geopolymer G-FA (**A**,**C**) and GSyl-FA (**B**,**D**). The scale bar is 1 mm.

Figure 5 shows the 2D and 3D microtomography of slices of the G-FA and GSyl-FA samples, respectively, while Figure 5 reports the corresponding pore size distributions.

**Figure 6:**
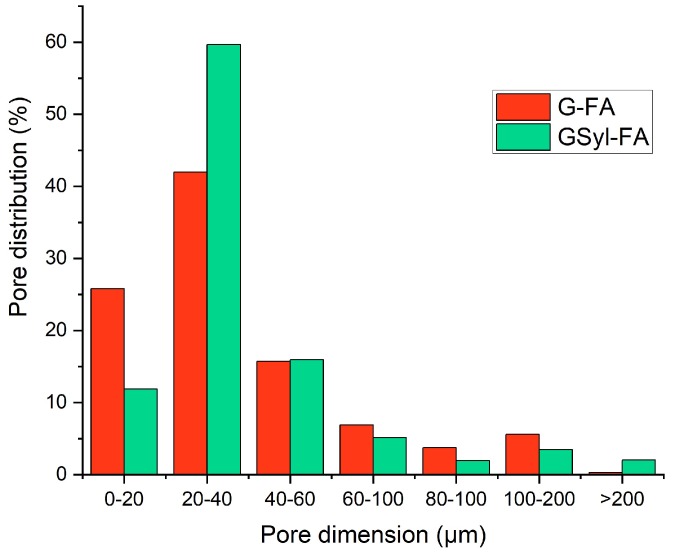
Pore size distribution in G-FA (red bars) and GSyl-FA (green bars) as obtained by an analysis of microtomography reported in Figure 5.

In agreement with SEM micrographs, microtomography images show that both systems were characterized by crack-free surfaces and a homogeneous bulk structure whose homogeneity is interrupted by the presence of not-reacted fly ash particles. The G-FA sample showed a total porosity of 7%, while the porosity of the GSyl hybrid sample was 20%, which could probably be ascribed to air bubbles entrapped in the slurry during the mixing step and water evaporation that took place during the curing time. Moreover, both samples showed a very low closed porosity (6.42 × 10^−3^% for G-FA and 4.64 × 10^−2^% for GSyl-FA). These data are in qualitative agreement with density measurements for these two specimens—1.70 g/cm^3^ in the case of G-FA and 1.56 g/cm^3^ in the case of GSyl-FA. In addition, the pore size distribution appeared rather similar in the two samples (Figure 6): Both samples had ≈90% of pores sized around 60 μm; G-FA had ≈27% of pores sized between 0 and 20 μm, and ≈40% of the pores sized between 20 and 40 μm; while GSyl-FA had ≈12% of the pores sized between 0 and 20 μm and ≈60% of the pores sized between 20 and 40 μm. As far as the ≈10% of the pores sized over 60 μm, the pores with highest dimension were sized 120 μm for G-FA, while an appreciable fraction of pores of for GSyl-FA was characterized by a diameter equal or greater to 200 μm (consistent with images reported in Figure 5). This different morphological feature in which the total porosity and the dimension of the pores was more pronounced in the case of the GSyl-FA specimen in respect to G-FA could be attributable the presence of polysiloxane units in GSyl-FA that could change: (1) The evaporation mechanism of the water molecules within geopolymer matrix during the curing process by bringing the molecules closer together before they evaporate, thus leaving larger voids; and (2) the increased viscosity of the hybrid paste in respect to unmodified geopolymer slurry [16] that prevents the air bubbles trapped in the mixture during the preparation process of the samples to easily escape. 

### 2.4. Mechanical Properties 

**Figure 7:**
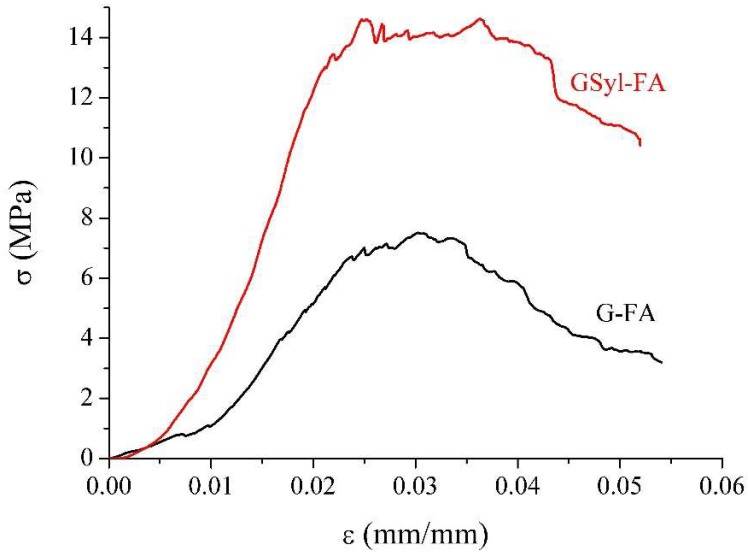
Stress–strain curves of the G-FA (black line) and GSyl-FA (red line) samples.

The mechanical properties of the unmodified FA-based geopolymer (G-FA) and of the corresponding hybrid sample containing 10% by weight of polysiloxane (GSyl-FA) prepared and cured in the same conditions are discussed in terms of uniaxial compressive stress–strain behavior, including compressive strength and Young’s modulus evaluation. Figure 7 shows the stress–strain curves up to failure. The resultant peak stress (compressive strength value) and the corresponding value of strain, as well as the ultimate failure strain (maximum strain of the samples) and the corresponding value of stress, are reported in Table 1. In regard to compressive strength, it is apparent that GSyl-FA was characterized by higher compressive strength than G-FA. In particular, GSyl-FA presented a noticeable increase (≈100%) of compressive strength compared to G-FA, although the hybrid sample was characterized by a lower density than the conventional one (1.70 g/cm^3^ in the case of G-FA and 1.56 g/cm^3^ in the case of GSyl-FA), a fact that by itself would have suggested a decrease in its mechanical properties. Similar considerations can be done by examining the values of the elastic moduli that, in the case of the hybrid sample, were doubled in respect to the conventional geopolymer. As previously discussed for metakaolin-based hybrids [16], this robust improvement of the mechanical properties was likely due to the presence of the polysiloxane component that was able to chemically interact with the inorganic matrix. This system was able to better absorb the applied load in terms of plastic deformation with respect to an unmodified geopolymer. As apparent from Figure 7 in the case of G-FA, once the maximum load value σc was reached (ε ≈ 3%), the internal micro-cracking rapidly spread and caused a progressive reduction in stiffness that coincided with the descending branch in the stress–strain curve, and a brittle rupture of the specimen occurred; in the case of GSyl-FA, once the maximum load value σ_c_ was reached (ε ≈ 2.5%), the specimen was able to absorb the maximum load by means of a further “plastic” deformation (corresponding to the branch of the curve at almost null slope) up ε ≈ 4%, when the final collapse took place.

Moreover, to the best of our knowledge, although the compressive strength values reached by the hybrid specimens presented in this paper were not very high (≈15 MPa), thus suggesting only non-structural applications such as repair mortars or prefabricated elements, an improvement of ≈100% of the mechanical properties in respect to the unmodified geopolymer obtained according the same experimental procedure (≈7 MPa) could be considered an unprecedented result in terms of the effectiveness of the applied synthetic strategy that was specifically aiming at obtaining hybrid materials with improved mechanical properties in respect to conventional ones. It is worth pointing out that in the only other case reported in the literature claiming the obtainment of a hybrid geopolymer [26], the improvement of the mechanical properties of the modified geopolymer was only around 25% in respect to the unmodified one [26]. 

## 3. Materials and Methods 

### 3.1. Materials 

Class F coal fly ash “EFA-Füller HP” (FA), whose composition is reported in Table 2, was supplied by BauMineral GmbH, Herten (Germany). Sodium hydroxide of reagent grade was supplied by Sigma-Aldrich (St. Louis, Missouri, USA). The sodium silicate solution (see composition in Table 2) was supplied by Prochin Italia S.r.l (Caserta, Italy). A commercial oligomeric dimethylsiloxane mixture was purchased from Globalchimica S.r.l (Turin, Italy) with the name of Globasil AL20.

### 3.2. Specimen Preparation

#### 3.2.1. Preparation of Unmodified Fly Ash-Based Geopolymers (G-FA)

The alkaline activating solution was prepared by mixing the sodium silicate solution with a 12 M aqueous solution of sodium hydroxide. The solution was then allowed to equilibrate and cool for 24 h. The composition of the obtained solution can be expressed as Na_2_O 1.21SiO_2_ 11.09H_2_O. Then, fly ash was incorporated into the activating solution with a liquid-to-solid ratio of 0.5:1 by weight and mixed by a mechanical mixer for 10 min at 400 rpm. The composition of the whole geopolymeric slurry can be expressed as Al_2_O_3_–4.65SiO_2_–0.78Na_2_O–7.78H_2_O, assuming that geopolymerization occurred at 100% (see discussion in Section 3.1). This system was assumed as the “unmodified” FA-based geopolymer material (and indicated in the following as G-FA). As soon as prepared, the specimens were cast into cubic molds and cured in >95% relative humidity conditions at 60 °C for 24 h. Subsequently, the specimens were kept at room temperature for a further 6 days in >95% relative humidity conditions and then for a further 21 days in open air. The apparent density of G-FA samples, as determined by hydrostatic balance, was 1.70 g/cm^3^.

#### 3.2.2. Preparation of Fly Ash-Based Hybrid Geopolymers (GSyl-FA)

Hybrid fly ash-based polysiloxane-geopolymer samples (GSyl-FA) were prepared by incorporating 10 wt % of a commercial oligomeric dimethylsiloxane mixture into the freshly prepared FA-based geopolymer suspension obtained according to the procedure described in the previous paragraph. The paste was then mixed by mechanical stirring for 10 min at 400 rpm. As soon as prepared, the specimens were cast into cubic molds and cured in >95% relative humidity conditions at 60 °C for 24 h. Subsequently, the specimens were kept at room temperature for a further 6 days in >95% relative humidity conditions and then for a further 21 days in open air. The apparent density of the GSyl-Fa sample, as determined by hydrostatic balance, was 1.56 g/cm^3^.

### 3.3. Methods

#### 3.3.1. Physical and Microstructural Assessment

The microstructure of all samples was assessed by SEM analysis and carried out by means of a Nova NanoSem 450 FEI microscope. The internal structure of the samples was studied by micro-computed tomography (µCT). These analyses were carried out with a µCT scanner (Skyscan 1272, Bruker, Kontich, Belgium) made available by the ATeN Center, University of Palermo (Palermo, Italy). The samples were scanned at a source voltage and current of 40 kV and 250 mA, respectively, with a total rotation of 180° and a rotation step of 0.2°. A 1 mm aluminum filter was chosen for the acquisitions. The image pixel size was 7.4 µm, and the scan duration was about 3 hours for every sample. The reconstructions were carried out using the software NRecon (Micro Photonics Inc, Allentown, PA, USA; version 1.6.10.2) starting from the acquired projection images. The 2D-images had a color depth of 8 bit with 265 grey level. After that, the whole set of raw images was displayed in a 3D space by a software CTVox. Quantitative analyses were carried out via the CTan software (version 1.16.1.0, Bruker, Billerica, Massachusetts, USA). These measurements allowed for the evaluation of the porosity of the samples.

Fly ash particle size distribution was determined by means of a Malvern Mastersizer 3000 laser particle analyzer (Malvern, Malvern, UK). 

Hydrostatic weighing for the apparent density measurements of the cured samples was carried out by means of a balance OHAUS-PA213 provided by Pioneer. 

#### 3.3.2. Compressive Behavior 

Uniaxial compressive tests were carried out on 50 × 50 × 50 mm^3^ cubic specimens by means of an MTS 810 servo-hydraulic universal testing machine (MTS System S.R.L., Torino, Italy). For each sample type, four specimens were tested under displacement control in order to obtain the corresponding stress–strain curve, compressive strength, and Young’s modulus. The compressive tests were performed until the sample ruptured at a constant displacement velocity of 0.20 mm/min. The measurement of the displacement was given by the crosshead displacement, while the Young’s modulus of each sample was computed from the linear stress–strain response recorded during the test. The values reported are the averages of the three compressions strength values. 

#### 3.3.3. X-Ray Diffraction Characterization

Wide angle X-ray diffraction patterns were obtained at room temperature (≈22 °C) with an automatic Rigaku powder diffractometer Miniflex 600 (Tokyo, Japan) operating in the θ/2θ Bragg–Brentano geometry and using CuKα radiation. The phase recognition was carried out by using the PDF-4+ 2014 (International Centre for Diffraction Data®, Newtown Square, Pennsylvania, USA) database and the Rigaku PDXL2 software (Rigaku, Tokyo, Japan).

## 4. Conclusions

In this paper, the preparation and characterization of new fly ash-based geopolymer hybrid materials are presented for the first time by extending a synthetic methodology developed for metakaolin-based materials standing on the simultaneous reactions of the polymerization and co-reticulation of both organic and inorganic networks. In particular, a commercial oligomeric dimethylsiloxane mixture was dispersed with FA in a silicate aqueous solution at room temperature (≈22 °C) and allowed to react. In these conditions, both the polycondensation reaction of FA and the polymerization of siloxane units took place at the same time, thus allowing for the formation of chemical links between the reactive FA-based geopolymeric suspension and the dimethylsiloxane mixtures. To the best of our knowledge, these are the only class II hybrid geopolymers obtained from fly ash and silicones thus far reported in the literature.

The hybrid materials obtained were characterized in terms of WAXD, SEM and X-ray microtomography which described an amorphous structure characterized by a diffuse porosity that was around 20% in the hybrid sample and in which, even at nanometric level, the organic and inorganic domains appeared to be indistinguishable. 

Mechanical characterization also showed that the dispersion of dimethylsiloxane units in the inorganic matrix enabled the enhancement of the compressive strength and elastic modulus of the hybrid organic-inorganic geopolymeric material up to 100% in comparison to the unmodified geopolymeric material obtained according to the same experimental procedure. This relative enhancement is unprecedented in the literature for similar materials.

For final considerations, it is worth pointing out that the described synthetic approach could also be applied to different types of low cost aluminosilicate sources (such as bottom ash, rice husk, blast furnace slag, sediments or muds). This suggests the possibility to produce geopolymer hybrids by reusing and valorizing waste raw resources and by-products, thus representing an effective contribution to the circular economy. 

Finally, as an immediate development of the present study, we aim at producing foamed fly ash-based hybrid materials for possible applications in the field of thermal and acoustic insulation, and, in order to further improve the mechanical properties of the final products, mortars, and concrete systems will be prepared. 

In addition, ongoing studies and investigations aimed at improving the reactivity of fly ash by the means of mechano–chemical grinding and/or thermal activation.

## Figures and Tables

**Table 1:** Compressive strength at peak stress (σ_c_), strain at peak stress (ε_c_), ultimate failure strain (ε_ult_), compressive stress at ultimate failure strain (σ_ult_), and Young’s modulus (E_c_) of the FA-based unmodified geopolymer (G-FA) and FA-based hybrid geopolymer (GSyl-FA) samples after 28 days of curing at ≈22 °C.

Samples	σ_c_ (MPa)	ε_c_ (%)	σ_ult_ (MPa)	ε_ult_ (%)	E_c_ (MPa)
**G-FA**	7.5 ± 0.2	3.0 ± 0.5	3.2 ± 0.2	5.4 ± 0.5	(1.8 ± 0.2) × 102
**GSyl-FA**	14.6 ± 0.1	3.2 ± 0.5	10.5 ± 0.5	5.2 ± 0.5	(4.0 ± 0.3) × 102

**Table 2:** Chemical composition (weight%) of the fly ash and sodium silicate solution used in this paper.

Fly Ash									
**Al_2_O_3_**	**SiO_2_**	**K_2_O**	**Fe_2_O_3_**	**Na_2_O**	**MgO**	**CaO**	**SO_3_**	**TiO_2_**	**LOI**
21.71	48.59	2.11	8.03	1.06	2.40	7.30	0.80	1.00	7.00
**Sodium Silicate Solution**									
**SiO_2_**	**Na_2_O**	**H_2_O**							
29.45	14.75	55.8							
